# Supplementary material for: The Oxygen and Glucose Deprivation of Immature Cells of the Nervous System Exerts Distinct Effects on Mitochondria, Mitophagy, and Autophagy, Depending on the Cells’ Differentiation Stage
Source: Brain Sci. 2023 Jun 4;13(6):910. doi: 10.3390/brainsci13060910 (PMC10296522; doi:10.3390/brainsci13060910)

FUNDC1

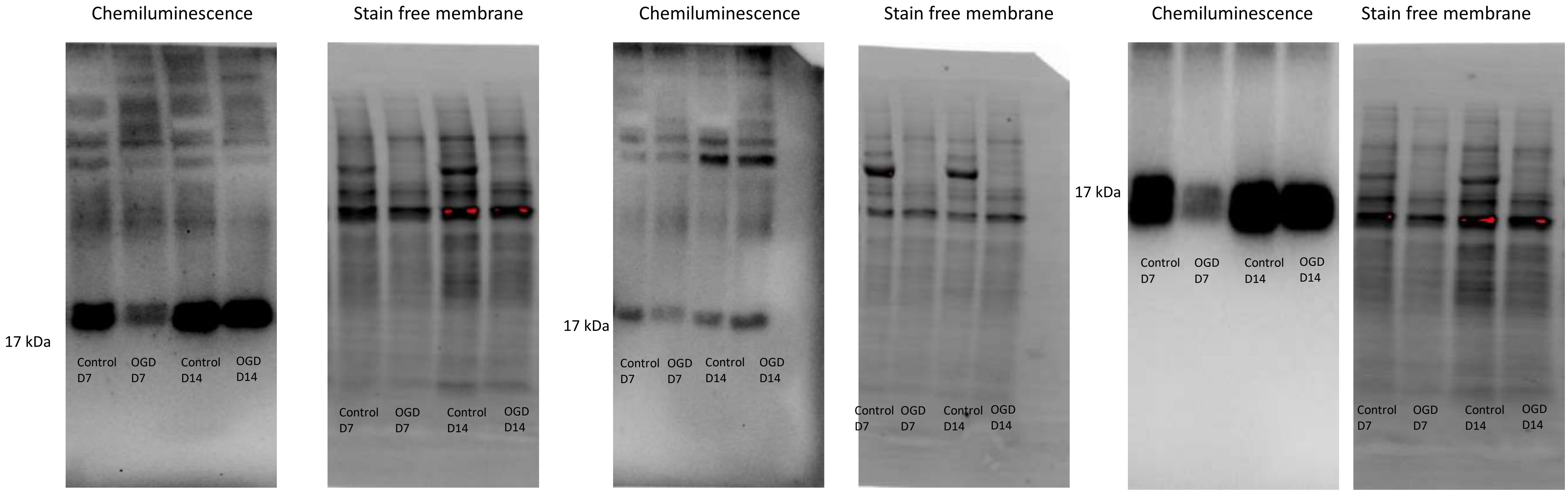
Chemiluminescence
17 kDa
Control D7
OGD D7
Control D14
OGD D14
Stain free membrane
Control D7
OGD D7
Control D14
OGD D14
Chemiluminescence
17 kDa
Control D7
OGD D7
Control D14
OGD D14
Stain free membrane
Control D7
OGD D7
Control D14
OGD D14
Chemiluminescence
17 kDa
Control D7
OGD D7
Control D14
OGD D14
Stain free membrane
Control D7
OGD D7
Control D14
OGD D14

PINK1

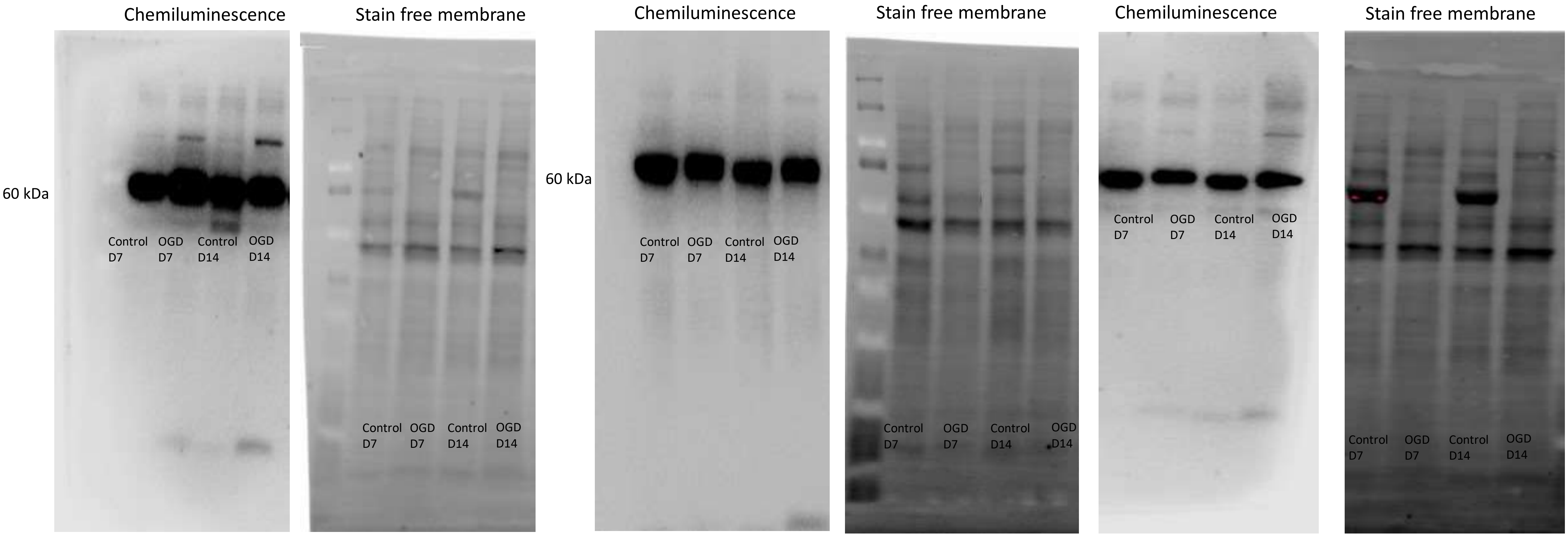
Chemiluminescence
Stain free membrane
Chemiluminescence
Stain free membrane
Chemiluminescence
Stain free membrane
60 kDa
Control D7
OGD D7
Control D14
OGD D14
Control D7
OGD D7
Control D14
OGD D14
60 kDa
Control D7
OGD D7
Control D14
OGD D14
Control D7
OGD D7
Control D14
OGD D14
Control D7
OGD D7
Control D14
OGD D14
Control D7
OGD D7
Control D14
OGD D14

p62

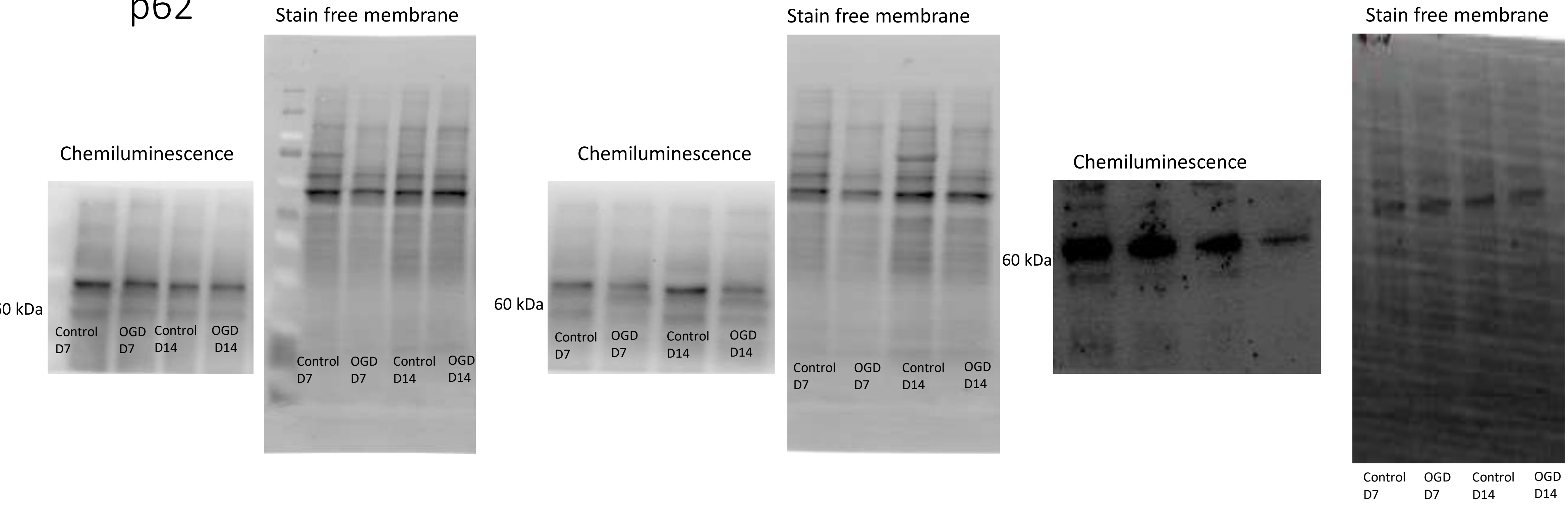

Chemiluminescence
Control D7
OGD D7
Control D14
OGD D14
Stain free membrane
Control D7
OGD D7
Control D14
OGD D14
Chemiluminescence
60 kDa
Control D7
OGD D7
Control D14
OGD D14
Stain free membrane
Control D7
OGD D7
Control D14
OGD D14
Chemiluminescence
60 kDa
Stain free membrane
Control D7
OGD D7
Control D14
OGD D14

LC3

Chemiluminescence

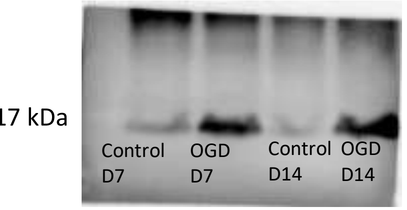

Stain free membrane

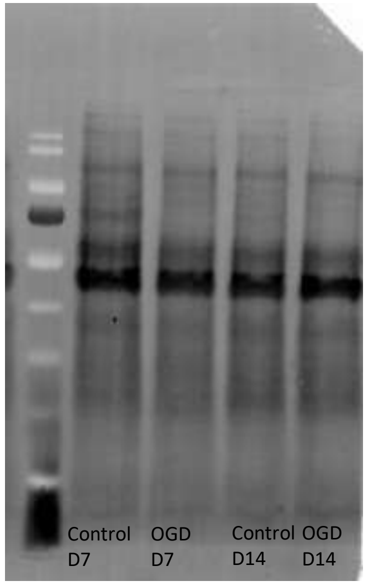

Chemiluminescence

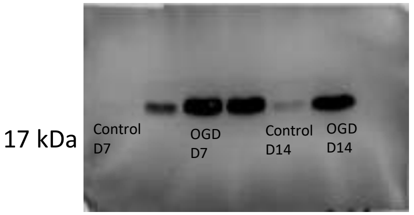

Stain free membrane

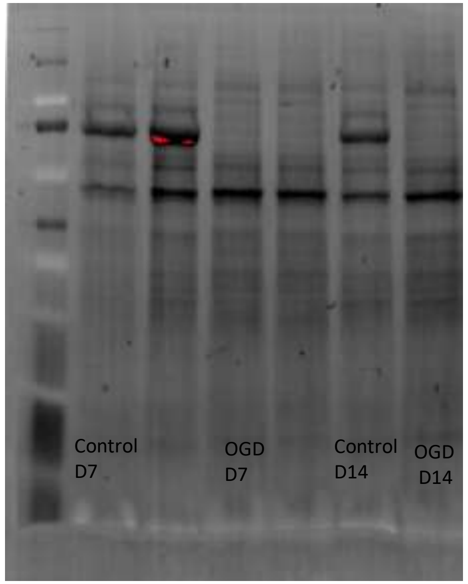

Chemiluminescence

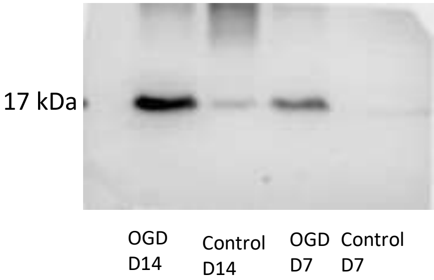

Stain free membrane

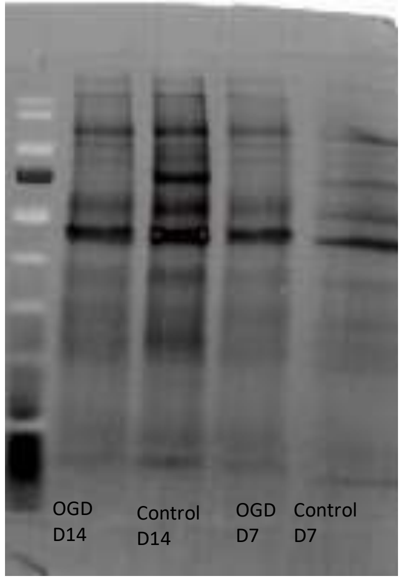

Supplement: Supplementary file 1 [file brainsci-13-00910-s001.zip › brainsci-2373970-supplementary.pdf]
